# Ib-M6 Antimicrobial Peptide: Antibacterial Activity against Clinical Isolates of *Escherichia coli* and Molecular Docking

**DOI:** 10.3390/antibiotics9020079

**Published:** 2020-02-12

**Authors:** J. M. Flórez-Castillo, P. Rondón-Villareal, J. L. Ropero-Vega, S. Y. Mendoza-Espinel, J. A. Moreno-Amézquita, K. D. Méndez-Jaimes, A. E. Farfán-García, S. Y. Gómez-Rangel, Oscar Gilberto Gómez-Duarte

**Affiliations:** 1 Facultad de Ciencias Exactas, Naturales y Agropecuarias, Ciencias Básicas Aplicadas para la Sostenibilidad—CIBAS, Universidad de Santander, Calle 70 No. 55-210, C.P. Bucaramanga (Santander) 680003, Colombia; jose.ropero@udes.edu.co; 2 Facultad de Ciencias de la Salud, Grupo de Investigación en Biología Molecular y Biotecnología, Universidad de Santander, Calle 70 No. 55-210, C.P. 680003 Bucaramanga (Santander), Colombia; 3 Facultad de Ciencias de la Salud, Grupo de Investigación en Manejo Clínico—CliniUdes, Universidad de Santander, Calle 70 No. 55-210, C.P. 680003 Bucaramanga (Santander), Colombia; buc14172051@mail.udes.edu.co (S.Y.M.-E.); julymoreno103@gmail.com (J.A.M.-A.); jkarendayana.1402@gmail.com (K.D.M.-J.); afarfan@udes.edu.co (A.E.F.-G.); sergio.gomez@udes.edu.co (S.Y.G.-R.); 4 Division of Pediatric Infectious Diseases, Department of Pediatrics, Jacobs School of Medicine and Biomedical Sciences, University at Buffalo, Buffalo, NY 14203, USA; oscargom@buffalo.edu

**Keywords:** antimicrobial peptides, *Escherichia coli*, molecular docking

## Abstract

The Ib-M6 peptide has antibacterial activity against non-pathogenic *Escherichia coli* K-12 strain. The first part of this study determines the antibacterial activity of Ib-M6 against fourteen pathogenic strains of *E. coli* O157:H7. Susceptibility assay showed that Ib-M6 had values of Minimum Inhibitory Concentration (MIC) lower than streptomycin, used as a reference antibiotic. Moreover, to predict the possible interaction between Ib-M6 and outer membrane components of *E. coli*, we used molecular docking simulations where FhuA protein and its complex with Lipopolysaccharide (LPS–FhuA) were used as targets of the peptide. FhuA/Ib-M6 complexes had energy values between −39.5 and −40.5 Rosetta Energy Units (REU) and only one hydrogen bond. In contrast, complexes between LPS–FhuA and Ib-M6 displayed energy values between −25.6 and −40.6 REU, and the presence of five possible hydrogen bonds. Hence, the antimicrobial activity of Ib-M6 peptide shown in the experimental assays could be caused by its interaction with the outer membrane of *E. coli*.

## 1. Introduction

The discovery of antibiotics in the 1940s is considered the most significant advance of medicine in the 20th century since it allowed the treatment of common but deadly diseases such as tuberculosis and pneumonia [1,2]. Simultaneously and inevitably, a natural phenomenon produced by the defense mechanisms of microorganisms known as antimicrobial resistance (AMR) arose. Moreover, it has presented a disturbing increase, mainly caused by the indiscriminate use of antibiotics. For this reason, the difficulty in the treatment of infections has increased. Similarly, the cost and risk of medical and surgical procedures has increased [3]. According to the Department of Health of the United Kingdom, by 2050, the number of deaths per year in the world caused by AMR could reach 10 million [2]. Due to this, AMR is recognized as a public health problem worldwide [3].

According to the World Health Organization (WHO), *Escherichia coli* is recognized as one of the most resistant bacteria [4], generating complications in the treatment of genitourinary tract infections, sepsis and meningitis acquired in the community or hospital stays [5].

To combat the problems mentioned above, WHO recommends increasing awareness of the antimicrobial resistance through communication and education. Furthermore, it is necessary to optimize the use of antimicrobials in human health as well as encouraging the development of new antimicrobial compounds. Therefore, antimicrobial peptides (AMPs) have attracted attention in the last three decades.

AMPs are part of the immune system of living organisms and have a broad spectrum of action against various pathogenic microorganisms, such as bacteria, fungi, and parasites, among others. Structurally, AMPs have a size ranging between 12 and 50 amino acids, and, in the great majority, have a positive charge and adopt amphipathic structures [6].

Although AMPs have a great variety of mechanisms of action, they have their cationic nature in common, which allows them to interact with the polyanionic cell membranes of microorganisms [7,8,9]. The electrostatic interaction depends on the cell and occurs with different components of the membrane. In the case of *E. coli*, this interaction occurs with lipopolysaccharides [10]. Some peptides like indolicin can form pores that allow the entry of the peptide into the cell. In another case, these pores can produce irreversible damage to the membrane, as in the case of the cepropins [11].

On the other hand, AMPs may bind to specific protein targets of the cell membrane. For example, the Microcin J25 binds to the FhuA protein, which is an important iron transporter in Gram-negative bacteria such as *E. coli*. Once attached to the protein, it can enter the cell to reach its final target, RNA polymerase [12]. FhuA protein is a crucial target of several antibiotics such as rifamycin and its derivatives as well as albomycin. In addition, it is the primary recipient of bacteriophages such as T1 and T5, among others. For this reason, it is considered an essential target for the development of new drugs. Additionally, it has been used for the design of specific antigens that can be used as vaccines against *E. coli* in the future [13].

Recently, a family of peptides named Ib-M with a high positive charge due to ARG residues and high hydrophobicity by TRP residues was synthesized. These peptides exhibited antibacterial activity against *E. coli* K-12 [14]. Among them, Ib-M6 showed the highest antibacterial activity with an IC50 value of 1 µM. The authors suggested that the high positive charge of the peptide facilitates the interaction with lipopolysaccharides of the membrane of *E. coli*. Moreover, the presence of TRP residues can favor the insertion in the cell. These phenomena can be determinant in its antimicrobial activity. Despite the above, the strain of *E. coli* used was non-pathogenic. Therefore, it is necessary to know the antibacterial properties of Ib-M6 in pathogenic strains in order to explore its potential application in in vivo models.

Having in mind the above, in this research, we report the antibacterial activity of Ib-M6 against 14 clinical isolates of *E. coli* from children under five years old from Bucaramanga, Colombia. Moreover, to have a first approximation about its interaction of this peptide with the surface of *E. coli*, molecular docking simulations were used to study the possible interaction of Ib-M6 with FhuA protein and its complex formed with lipopolysaccharide (LPS). Our results suggest that the antibacterial activity shown by the Ib-M6 peptide against pathogenic *E. coli* strains could be caused by its interactions with LPS.

## 2. Results

### 2.1. Antimicrobial Activity

The Minimum Inhibitory Concentration (MIC) of Ib-M6 peptide was determined against *E. coli* clinical and non-pathogenic isolates. In Table 1, MIC and Minimal Bactericide Concentration (MBC) values are shown.

The Ib-M6 peptide inhibited the growth of *E. coli* in a range of 0.7 to 25 μM for pathogenic strain, and a range of 1.5 to 3.1 μM for non-pathogenic *E. coli*. The lowest and highest MIC values were observed in EAEC_2, and EIEC strains with 0.7 µM and 25 µM, respectively. In addition, MIC values were different between the same pathotype, and in comparison with other pathotypes. Testing the reference drug streptomycin, MIC values between 6.25 μM and 200 μM for the pathogenic strains, and values between 3.1 to 400 μM for the non-pathogenic strain, were obtained. The MIC values of Ib-M6 were lower than those obtained for the reference antibiotic, except for ETEC_2 strains. In most of the strains evaluated, MBC values were higher than those observed in the MIC tests.

### 2.2. Circular Dicrhroism and 3D Structure

**Figure 1:**
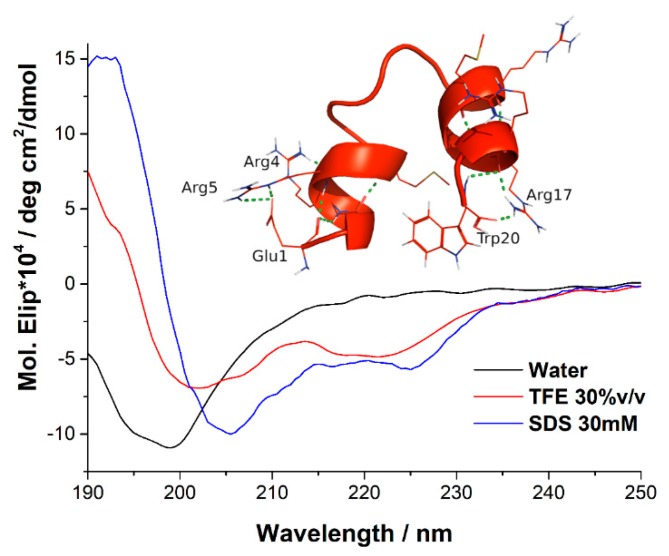
Circular dichroism spectra of Ib-M6 in water (black line), 30 mM of sodium dodecyl sulfate (SDS) (blue line), and 30% trifluorethanol (TFE) (red line). The spectra were obtained at a peptide concentration of 106 µM at room temperature. (Insert: Structure of the best model of the peptide obtained by PepFold).

The secondary structure of Ib-M6 was determined by circular dichroism (CD). For this, we used membrane mimicking environments. The TFE (trifluorethanol) was used for mimicking the hydrophobic microbial membrane, and SDS (sodium dodecyl sulfate) was used for the negatively charged prokaryotic membrane. In Figure 1, the circular dichroism spectra of Ib-M6 in water, 30% TFE, and 30 mM sodium dodecyl sulfate SDS, are shown.

The CD spectra indicated that Ib-M6 presented a strongly negative peak in the water at approximately 198 nm, which is characteristic of a random structure. On the other hand, two minima at approximately 206 and 226 nm in the presence of SDS were observed. Additionally, in TFE, this negative peak appears in 201 and 221 nm. This behavior can be related to a helical structure of the peptide in both TFE and SDS.

The three-dimensional structure was determined using the PepFold server (insert Figure 1). As can be seen, the N and C-terminal sections present alpha-helix structures stabilized by hydrogen bonds between the NH and CO groups of the peptide bond every four residues. In addition, interactions between the side chains of the residues of GLU1, ARG5, TRP20, and ARG17 occur. On the other hand, it is observed that the central region of the peptide has a loop structure due to the presence of GLY in positions 8, 10, and 12.

### 2.3. Electrostatic Potential Map of Ib-M6 Peptide and FhuA Protein

**Figure 2:**
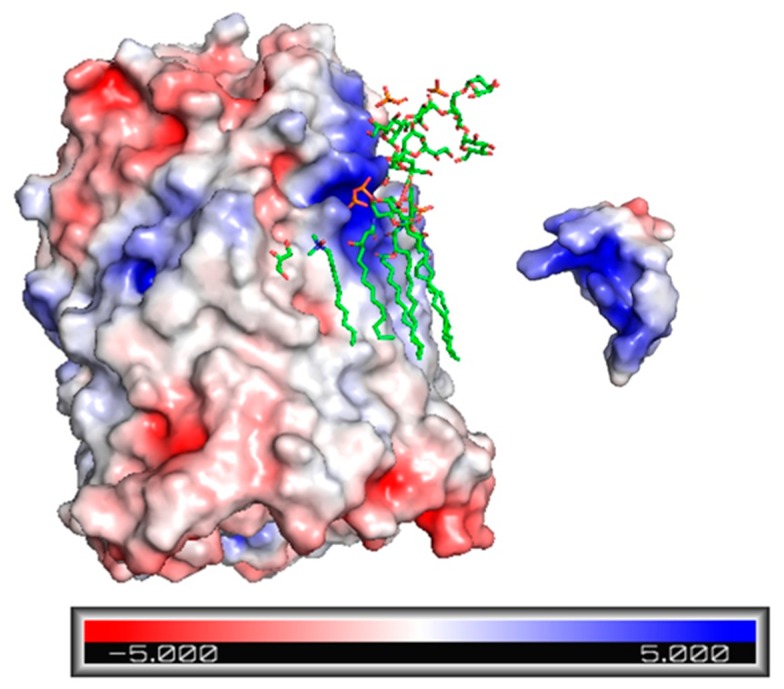
Electrostatic potential maps of FhuA protein (left) and Ib-M6 (right). Blue areas are those with high potential and with a relative absence of electrons (positive charge). Red areas are those with low potential and with a relative abundance of electrons. The other areas between red and blue are those with a neutral charge.

To visualize possible regions of interaction between Ib-M6 peptide and FhuA protein, the electrostatic potential maps of both molecules were obtained in Pymol. As shown in Figure 2, Ib-M6 peptide has a great positive charge area (blue) and could interact with the upper region of FhuA that are colored in red. Moreover, the neighborhood nearby the LPS has a positive charge in the upper part and a neutral charge in the lower part. Hence, if Ib-M6 peptide interacts with LPS, it is likely that it would be in the lower part.

### 2.4. Docking Results for the Ib-M6 Peptide in Complex with FhuA Protein

The three-dimensional structure of the peptide Ib-M6 using the PepFold server was predicted, and the five best models were selected to perform the molecular docking simulations. These models were named as M6_A, M6_B, M6_C, M6_D, and M6_E. For example, Figure 1 (insert) shows the structure of the best PepFold model (named in this study as M6_A) in terms of the lower value of the template model (TM) score, which determines the structural similarity between one structure of the PepFold database and the modeled structure.

For each of the five models obtained for Ib-M6 peptide, two molecular docking simulations were performed. First, the peptide was allowed to move around the FhuA protein (global docking), and the best model in terms of the interface score (I_sc) was selected to perform a new molecular docking simulation to refine the obtained complex.

Table 2 shows the I_sc values of the selected complexes in global docking simulations. FhuA+M6_E had the lowest value of I_sc. Hence this is the most stable complex obtained in global docking simulations.

**Figure 3:**
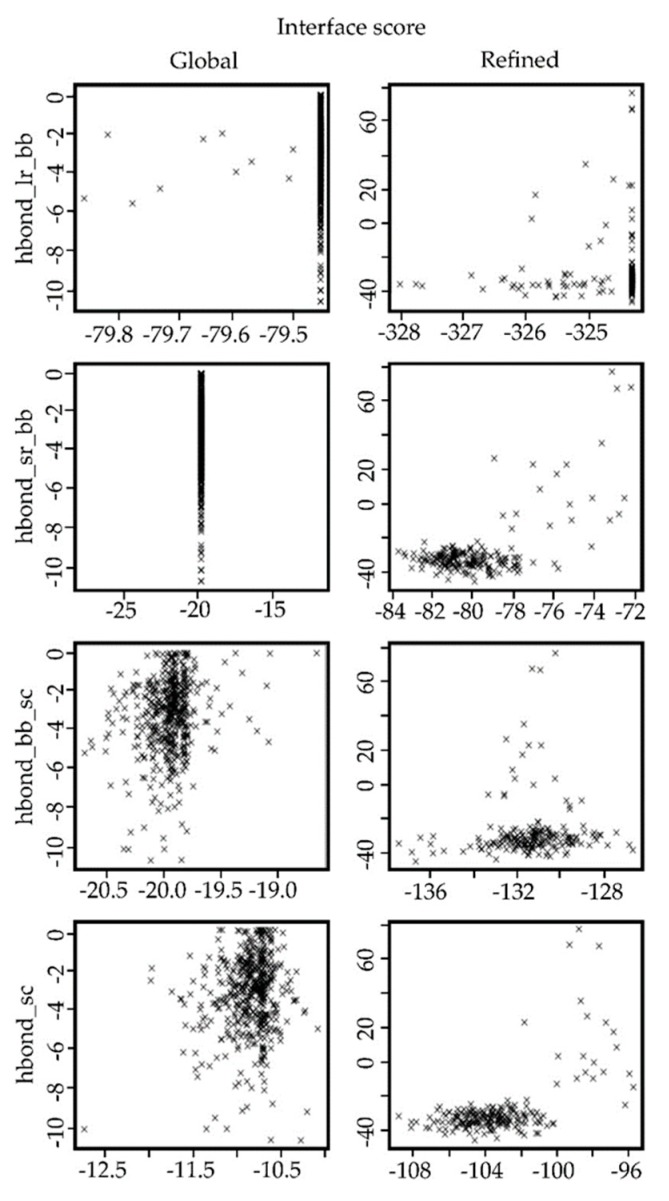
Comparison of different terms in the score file registered for the complexes created using the global protein–protein protocol versus the values obtained for the models created using the FlexPepDock protocol. hbond_lr_bb = energy of long-range hydrogen bonds (kcal/mol), hbond_sr_bb = energy of short-range hydrogen bonds (kcal/mol), hbond_bb_sc = energy of backbone–side-chain hydrogen bonds (kcal/mol), and hbond_sc = energy of side-chain–side-chain hydrogen bonds (kcal/mol).

In Figure 3, the scatterplots of the models obtained in each step of the docking procedure are shown. The top row shows the values obtained for the predicted models in the global protein–protein protocol. In contrast, the bottom row shows the values obtained for the predicted models in the refinement process using the FlexPepDock protocol.

In Table 3, I_sc values of the three best-refined complexes selected per each peptide model are shown. Moreover, the possible hydrogen bonds found in the selected refined complexes are also included. As seen in Table 3, three complexes between FhuA and model M6_B of the peptide (FhuA+M6_B_1 to FhuA+M6_B_3) show a hydrogen bond between TYR307 of FhuA and GLY8 of the peptide with different lengths. The interface score values of these three models are around –33 Rosetta Energy Units (REU). Similarly, two complexes between FhuA and model M6_A (FhuA+M6_A_1 and FhuA+M6_A_2) showed a hydrogen bond between GLY548 and ARG14 with lengths higher than 2 Å, and interface score values around −41 REU.

**Figure 4:**
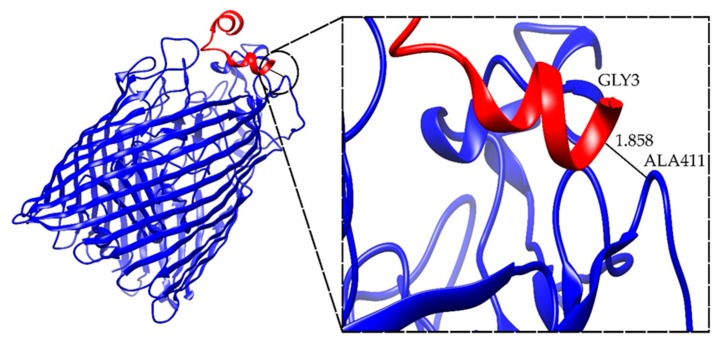
Structure of FhuA+M6_A_1 complex. The images of the remaining selected complexes can be found in the supplementary information.

In Figure 4 the complex FhuA+M6_A_1 which has the best interface score value is shown.

### 2.5. Docking Results for the Ib-M6 Peptide Interacting with Lipopolysaccharide–FhuA Complex

In Table 4, the interface score for the models between LPS–FhuA and the five Ib-M6 peptide models obtained in global docking simulations are shown. LPS–FhuA+M6_A has a more negative value of I_sc than the values obtained for the complexes with the other peptide models. Hence, this is the best complex found in these docking simulations.

**Figure 5:**
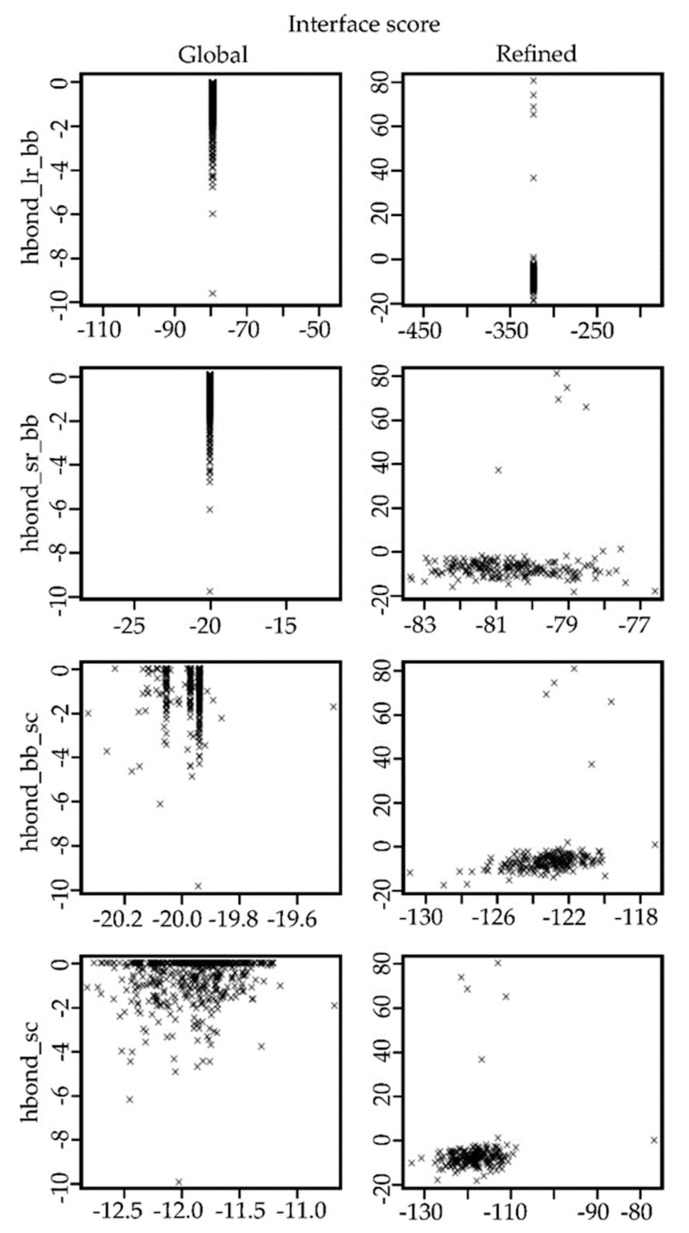
Comparison of different terms in the score file registered for the complexes created using the global protein–protein protocol versus the values obtained for the models created using the FlexPepDock protocol. hbond_lr_bb = energy of long-range hydrogen bonds (kcal/mol), hbond_sr_bb = energy of short-range hydrogen bonds (kcal/mol), hbond_bb_sc = energy of backbone–side-chain hydrogen bonds (kcal/mol), and hbond_sc = energy of side-chain–side-chain hydrogen bonds (kcal/mol).

In the same way, as with the FhuA protein, five complexes between LPS–FhuA and Ib-M6 peptide were selected from the global docking simulations to perform the refined molecular docking. In Figure 5, the comparisons of different terms in the scoring function of Rosetta for both docking scenarios are shown. The images of the remaining complexes are available in the supplementary information (S.1.). As expected, the energy values for the refined complexes were better than the values for the global complexes.

For the refined complexes between the LPS–FhuA and Ib-M6 peptide models, multiple possible hydrogen bonds are found in each complex (Table 5).

**Figure 6:**
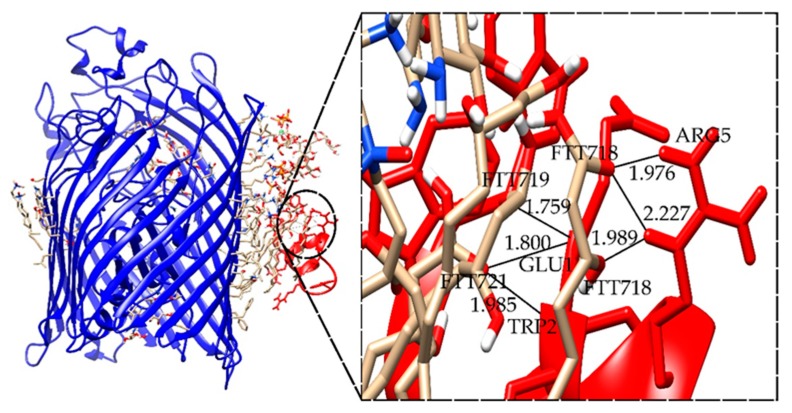
Structure of LPS–FhuA+M6_B_1 complex. The images of the remaining selected complexes can be found in the supplementary information.

Figure 6 shows the complex LPS–FhuA+M6_B_1, which has the best interface score. Moreover, the possible hydrogen bonds between Lipid A of LPS and Ib-M6 peptide are shown. The images of the remaining complexes are available in the supplementary information (S.4.).

Although our results suggest that Ib-M6 peptide interacts better with LPS than with FhuA protein, further in vitro complementary tests should be performed to confirm the interaction with the cell membrane of *E. coli*.

## 3. Discussion

The Ib-M6 peptide has been shown to have antimicrobial activity against *E. coli* K-12, a non-pathogenic strain. Furthermore, it has no hemolytic activity against human erythrocytes. This makes it a possible candidate for a new antibiotic against infections caused by these bacteria. The strains of *E. coli* evaluated in this study were sensitive to the Ib-M6 peptide with MIC values between 0.7 and 25 µM. Similarly, strains EPEC_2, DAEC_1, EIEC, and the non-pathogenic strains_1 and 3 presented a higher streptomycin resistance with MIC values between 100 and 400. However, these strains were sensitive to peptide Ib-M6. Those differences in MIC values could be explained by virulence factors presented in pathogenic strains or by different behaviors of strains using in vitro cultures. Ebbensgaard et al. reported variations in MIC values between 1 to 16 μg/mL testing eight mutants obtained from one clinically isolated *E. coli* [15]. Indeed, current evidence suggests that the inclusion in future studies of several strains from all different pathotypes should be considered. MBC was obtained in a range of 1.5 to 50 μM. The Ib-M6 activity was considered bactericide in most of the strains included, except EAEC_1, ETEC_2, and non-pathogenic_2 strains. STEC_2 and non-pathogenic_3 strains showed the same MIC and MBC results. The effect in the ETEC_2 strain was considered bacteriostatic because the MBC could not be obtained at evaluated concentrations.

CD assays suggest that the Ib-M6 peptide does not have a defined structure in a hydrophilic environment and acquires a stable three-dimensional structure in the presence of a hydrophobic environment.

The analysis of different terms in the score file given by Rosetta showed that the obtained refined models achieved better values than the models obtained in the global protein–protein docking. In the refinement protocol, the flexibility of the peptide is included. This form allows a better fitting of the small molecule in the receptor structure. Hence, the obtained results were expected.

For the simulations focused on FhuA protein, the best complex has one hydrogen bond between ALA411 of FhuA and GLY3 of peptide Ib-M6 with a length of 1.858 Å, which implies a stable interaction. Moreover, all the complexes analyzed showed only one possible hydrogen bond. Thus, the binding of Ib-M6 with FhuA could not be stable enough.

It has been reported that the first step in the mechanism of action of PAMs is the electrostatic interaction between the positive charge of the peptide and the anionic membranes of bacteria [15,16]. In *E. coli*, this interaction is carried out with the lipopolysaccharide (LPS) present on the outer face of the membrane. The LPS has an orderly structure with low fluidity that determines permeability and prevents rapid diffusion of solutes through the membrane. The hydrophobic section of the LPS is constituted by lipid. That is a glucosamine based saccharolipid [17].

In our research, the simulations focused on LPS–FhuA multiple hydrogen bridges are observed between the 3-hydroxytetradecanoic residues, which are a structural part of lipid A, and the arginine residues of Ib-M6. The insertion of the peptide into the cell membrane is likely favored by Arg and Trp residues in Ib-M6. It has been reported that between these two amino acids, cation–π interactions are generated that make the insertion of Arg into the hydrophobic environment of the membrane more energetic [18]. These interactions do not prevent the Arg side chain from forming hydrogen bonds with the lipid A components. Our results suggest that it is possible that the Ib-M6 peptide inserts into the cell membrane of the bacteria for its internalization or pore formation. Further studies are required to determine the type of pores formed in order to establish their role on the antimicrobial activity of Ib-M6.

## 4. Materials and Methods

### 4.1. Antimicrobial Agents and Bacterial Strains

Ib-M6 peptide (sequence EWGRRMMGWGRGRRMMRRWW; MW = 2738.39 g/mol and charge 6+) was synthesized by Biomatik^®^ (Wilmington, DE, USA) with a purity of 98.5% and Streptomycin by Sigma (St. Louis, MO, USA).

Pathogenic strains of *Escherichia coli* were obtained from Laboratorio de Investigaciones Biomédicas y Biotecnológicas (LIBB) of Universidad de Santander. These strains were previously characterized, as described in a pilot case-control study [19]. A total of 14 strains were included in the study: 11 enteropathogenic isolates of *E. coli* and three non-pathogenic. Among the diarrheagenic pathotypes, two of each of the following were included: diffusely adherent *E. coli* (DAEC), enteroaggregative *E. coli* (EAEC), enteropathogenic *E. coli* (EPEC), enterotoxigenic *E. coli* (ETEC), and Shiga-toxin producing *E. coli* (STEC). Regarding enteroinvasive *E. coli* (EIEC), only one strain was included. To identify *E. coli* pathotypes, DNA template of five *E. coli* colonies per subject were tested for target genes with multiplex polymerase-chain reaction (mPCR). The mPCR was designed to detect genes found in six well-established pathogenic *E. coli* pathotypes as follows: gene daaE for DAEC; aaiC and aggR for EAEC; ipaH for EIEC; eae and/or bfpA for EPEC; lt and/or st for ETEC; and stx for STEC, as described before [19].

### 4.2. Antimicrobial Activity of Ib-M6 Peptide

For the Minimum Inhibitory Concentration (MIC) test, strains of *E. coli* were aerobically cultured for 18–20 h at 37 °C until the exponential growth phase was reached. For the microdilution method in broth at Mueller Hinton (MH), 100 µL two-fold dilutions of Ib-M6 peptide from 100 µM to 0.7 µM were added to 96-well polystyrene plates, 100 µL of *E. coli* inoculum adjusted to a concentration of 5 × 105 CFU/mL (5 × 104 CFU/well) were subsequently added and incubated for 18 h at 37 °C. Finally, the MIC was determined by direct observation and by reading at 620 nm on a spectrophotometer. Strains were tested in duplicate with its respective controls: untreated *E. coli* (positive growth control), negative control (bacteria-free MH medium), and streptomycin as reference antibiotic (serial dilutions from 400 to 1.5 µM). MIC was defined as the minimum concentration of the compound that allowed total inhibition of bacterial growth. For the Minimum Bactericidal Concentration (MBC), 100 µL were taken from the well where no growth was observed in the MIC plates and massive blood agar sowings were performed and incubated for 24 h at 37 °C. MBC was determined as the concentration of peptide at which 99.9% of bacteria were eliminated. MIC and MBC were developed according to The Clinical and Laboratory Standards Institute (CLSI) [20]. MIC and MBC were determined in triplicate and reported median values.

### 4.3. Circular Dichroism and Structure 3D of Peptide

Circular dichroism was used to examine the secondary structure of Ib-M6 peptide. The experiments were performed using a J 815 spectropolarimeter in a 10 mm light pass length quartz cell at 20 °C. The spectra were collected from 190 nm to 250 nm with a data pitch of 0.5 nm and a scan rate of 100 nm/min. The concentration of peptide was 106 µM in all cases and sodium dodecyl sulfate (SDS, 30 mM) or water were used as solvent. The structure of Ib-M6 peptide was constructed using the PepFold server [21,22]. Only the five best structural models (A–E) were used for the docking procedures.

### 4.4. Preparation of Ligand and Targets Molecules

The crystal structure of *Escherichia coli* proteins lipopolysaccharide and FhuA were derived from RCSB PDB Protein Data Bank (PDB ID 1QFG, https://www.rcsb.org/). Molecular docking was performed using the program Rosetta [23], which requires an initial PDB file that includes the molecules that are going to be docked. Hence, the user sponsored molecular visualization system Pymol [24] was used to create the necessary PDB files to run the molecular docking simulations.

### 4.5. Docking Procedure for the Ib-M6 Peptide in Complex with FhuA Protein

In this procedure for each Ib-M6 peptide model, a PDB file containing both peptide and FhuA protein was manually created using Pymol. Later, each of the five resulting PDB files was prepared using the cleanPDB Rosetta python script, to remove unknown atoms and water molecules.

Docking simulations were performed in two stages. In the first, 500 complexes were built for each peptide model by using a global protein–protein docking protocol, with both molecules treated as rigid. Later, the best model in terms of the interface score (I_sc) and location of the peptide in the iron recognition domain was selected from the resulting 500 complexes for each peptide model, i.e., five complexes were selected to be used in the second stage. For each of the selected models, the FlexPepDock Rosetta protocol was used to obtain 200 refined complexes by performing molecular docking simulations with a flexible peptide. Finally, for each peptide model, the best three resulting complexes were selected as the potential complexes between FhuA protein and Ib-M6 peptide. The protocols and flags used in the docking simulations are available in the supplementary information (S.1.).

### 4.6. Docking Procedure for the Ib-M6 Peptide in Complex with Lipopolysaccharide–FhuA

In this case, the initial complexes of the five peptide models were created manually in Pymol, and later treated with the clean_pdb_keep_ligand.py Rosetta python script to remove water molecules. In the same manner used for the FhuA protein, the five initial complexes were used to create 500 complexes for each peptide model by using the global protein–protein docking protocol. The best resulting complex for each peptide model was selected in terms of the Interface score (I_sc) and the proximity to the LPS. Later, the molecular docking simulations with flexible peptide were performed, and 200 complexes were created for each peptide model. In the end, the three best resulting complexes were selected as the potential complexes between LPS–FhuA and Ib-M6 peptide. The protocols and flags used in the docking simulations are available in the supplementary information (S.2.).

### 4.7. Analysis of the Resulting Potential Complexes

The selected complexes were analyzed in terms of the interface score (I_sc), the energy of long-range hydrogen bonds (hbond_lr_bb), the energy of short-range hydrogen bonds (hbond_sr_bb), the energy of backbone–side-chain hydrogen bonds (hbond_bb_sc), and the energy of side-chain–side-chain hydrogen bonds (hbond_sc), given by the scoring function of Rosetta [25]. The images of the possible hydrogen bonds between the molecules were obtained by using the extensible molecular modeling system UCSF Chimera [26].

## Figures and Tables

**Table 1:** The Minimum Inhibitory Concentration (median values, *n* = 3) and the minimum bactericide concentration (median values, *n* = 3) of Ib-M6 peptide on *Escherichia* *coli* strains.

Name Strain	MIC * (µM)		MBC * (µM)
	Ib-M6	Streptomycin	
EAEC_1	1.5	6.25	25
EAEC_2	0.7	6.25	1.5
ETEC_1	3.1	6.25	12.5
ETEC_2	12.5	6.25	> 100
EPEC_1	3.1	6.25	6.25
EPEC_2	1.5	100	3.1
DAEC_1	6.25	200	12.5
DAEC_2	3.1	50	6.25
EIEC	25	200	50
STEC_1	12.5	50	25
STEC_2	6.25	50	6.25
Non-pathogenic_1	1.5	200	3.1
Non-pathogenic_2	1.5	3.1	25
Non-pathogenic_3	3.1	400	3.1

* Standard deviation (SD) = 0.

**Table 2:** Interface score of the selected complex in global docking simulations for each peptide model.

Complex	I_sc (REU)
FhuA+M6_A	−8.928
FhuA+M6_B	−7.407
FhuA+M6_C	−8.334
FhuA+M6_D	−7.099
FhuA+M6_E	−10.305

**Table 3:** Summary of the interface score and hydrogen bonds present in the refined complexes between FhuA and peptide models. In the name of the complexes, the letters A, B, or D represent the selected model in the global docking stage, and numbers 1, 2, or 3 indicate the selected complexes in the refinement procedure.

Complex	I_sc (REU)	Hydrogen Bonds	Length (Å)
FhuA+M6_A_1	−42.545	ALA411-GLY3	1.858
FhuA+M6_A_2	−41.622	GLY548-ARG14	2.216
FhuA+M6_A_3	−41.466	GLY548-ARG14	2.277
FhuA+M6_B_1	−33.700	TYR307-GLY 8	1.849
FhuA+M6_B_2	−33.421	TYR 307-GLY 8	2.616
FhuA+M6_B_3	−33.071	TYR 307-GLY 8	2.363
FhuA+M6_D_1	−39.595	PRO415-GLU1	1.843

**Table 4:** Interface score of the selected complex in the global docking simulations for each peptide model.

Complex	I_sc (REU)
LPS–FhuA+M6_A	−6.038
LPS–FhuA+M6_B	−3.975
LPS–FhuA+M6_C	−3.772
LPS–FhuA+M6_D	−4.021
LPS–FhuA+M6_E	−4.833

**Table 5:** Summary of the interface score and hydrogen bonds present in the refined complexes between Lipopolysaccharide (LPS)–FhuA and Ib-M6 peptide models.

Complex *	I_sc (REU)	Hydrogen Bonds **	Length (Å)
LPS+M6_B_1	−40.625	FTT718-ARG5	1.976
		FTT718-ARG5	2.227
		FTT718-ARG5	1.989
		FTT719-GLU1	1.759
		FTT721-GLU1	1.800
		FTT721-TRP2	1.985

FTT = 3-hidroxy-tetradecanoic acid. * In the name of the complexes, the letters A, B, or D represent the selected model in the global docking stage, and numbers 1, 2, or 3 indicate the selected complexes in the refinement procedure. ** Hydrogen bonds between residue of FhuA (left) and residue Ib-M6 (right).

## Supplementary Materials

The following are available online at https://www.mdpi.com/2079-6382/9/2/79/s1. S1. Docking Simulations with the Software Rosetta1. Protocol for the simulations between FhuA and Ib-M6 peptide models. S2. Docking Simulations with the Software Rosetta1. Protocol for the simulations between LPS and Ib-M6 peptide models. S3. Images of the resulting complexes between FhuA and Ib-M6 peptide models. S4. Images of the resulting complexes between LPS and Ib-M6 peptide models.
