# Supplementary material for: Ib-M6 Antimicrobial Peptide: Antibacterial Activity against Clinical Isolates of Escherichia coli and Molecular Docking
Source: Antibiotics (Basel). 2020 Feb 12;9(2):79. doi: 10.3390/antibiotics9020079 (PMC7168133; doi:10.3390/antibiotics9020079)
Supplement: Supplementary file 1 [file antibiotics-09-00079-s001.pdf]

Supplementary Information

Docking Simulations with the Software Rosetta [1]

Protocol for the simulations between FhuA and Ib-M6 (EWGRRMMGWGRGRRMMRRWW-NH2) peptide models

Steps:

1. The crystallographic structure of the FhuA protein was obtained from the Protein Data Bank database with PDBid 1QFG.

2. Create initial FhuA-IbM6 complexes using Pymol and the different models of the Ib-M6 peptide.

3. Use the Rosetta python script: cleanPDB to remove unknown atoms, water molecules and to renumber the atoms of the complexes.

4. Run the protocol for protein-protein docking for an initial global docking process between protein FhuA and the peptide:

   docking_protocol.linuxgccrelease @**flag_global_docking**

   - Details of **flag_global_docking** file:

     -in:file:s input_files/complex.pdb
     -unboundrot input_files/complex.pdb

     -nstruct 500

     -partners A_B
     -dock_pert 3 8
     -spin
     -randomize1
     -randomize2

     -ex1
     -ex2aro

     -out:path:all output_files
     *-out:suffix _Model_*

5. Analize the best models in terms of I_sc and location of the peptide in the iron recognition domain.

6. Perform the refinement protocol using FlexPepDocking protocols

   - Perform a prepack step with the selected model, using the FlexPepDocking protocol with the file: **prepack_flags**

- Details of the **prepack_flags**

    -s input_files/best_model.pdb
    -ex1
    -ex2aro
    -use_input_sc
    -unboundrot input_files/best_model.pdb
    -flexpep_prepack
    -nstruct 1

- *Rename the obtained file as best_model_ppk.pdb*
- *Run the FlexPepDocking protocol with the file* ***flagsNoLow***

    - *Details of the file* ***flagsNoLow***

        -s input_files/best_model_ppk.pdb
        -out:pdb
        -out:path:all output_files
        -scorefile score.sc
        -nstruct 200
        -flexPepDocking:flexpep_score_only
        -flexPepDocking:pep_refine
        -ex1
        -ex2aro
        -unboundrot input_files/best_model.pdb
        -use_input_sc
        -mute protocols.moves.RigidBodyMover
        -mute core.chemical
        -mute core.scoring.etable
        -mute protocols.evalution
        -mute core.pack.rotamer_trials
        -mute protocols.abinitio.FragmentMover
        -mute core.fragment
        -mute protocols.jd2.PDBJobInputter

7. Analyze the results using Chimera and VMD.

Protocol for the simulations between LPS and Ib-M6 peptide models

1. The crystallographic structure of the FhuA protein was obtained from the Protein Data Bank database with PDBid 1QFG.

2. Create initial FhuA-Ib-M6 complexes using Pymol and the different models of the Ib-M6 peptide.

3. Use the Rosetta python script: clean_pdb_keep_ligand.py to remove water molecules and to renumber the atoms of the complexes.

4. Run the protocol for protein-protein docking for an initial global docking process between LPS and the peptide:

   docking_protocol.linuxgccrelease @**flag_global_docking**

   - Details of **flag_global_docking** file:

     -in:file:s input_files/complex.pdb
     -unboundrot input_files/complex.pdb

     -nstruct 500

     -partners A_B
     -dock_pert 1 1
     -spin
     -randomize2

     -ex1
     -ex2aro

     -out:path:all output_files
     *-out:suffix _Model_*

8. Analize the best models in terms of I_sc and the proximity to the LPS.

9. Perform the refinement protocol using FlexPepDocking protocols

   - *Run the FlexPepDocking protocol with the file* ***flagsNoLow***

     - *Details of the file* ***flagsNoLow***

       -s input_files/best_model.pdb
       -out:pdb
       -out:path:all output_files
       -scorefile score.sc
       -nstruct 200
       -flexPepDocking:flexpep_score_only
       -flexPepDocking:pep_refine
       -ex1
       -ex2aro
       -unboundrot input_files/best_model.pdb
       -use_input_sc
       -mute protocols.moves.RigidBodyMover
       -mute core.chemical
       -mute core.scoring.etable
       -mute protocols.evalution
       -mute core.pack.rotamer_trials
       -mute protocols.abinitio.FragmentMover
       -mute core.fragment

-mute protocols.jd2.PDBJobInputter

10. Analyze the results using Chimera and VMD.

Images of the resulting complexes between FhuA and Ib-M6 peptide models

Model FhuA-M6_A

- Model FhuA-M6_A_1

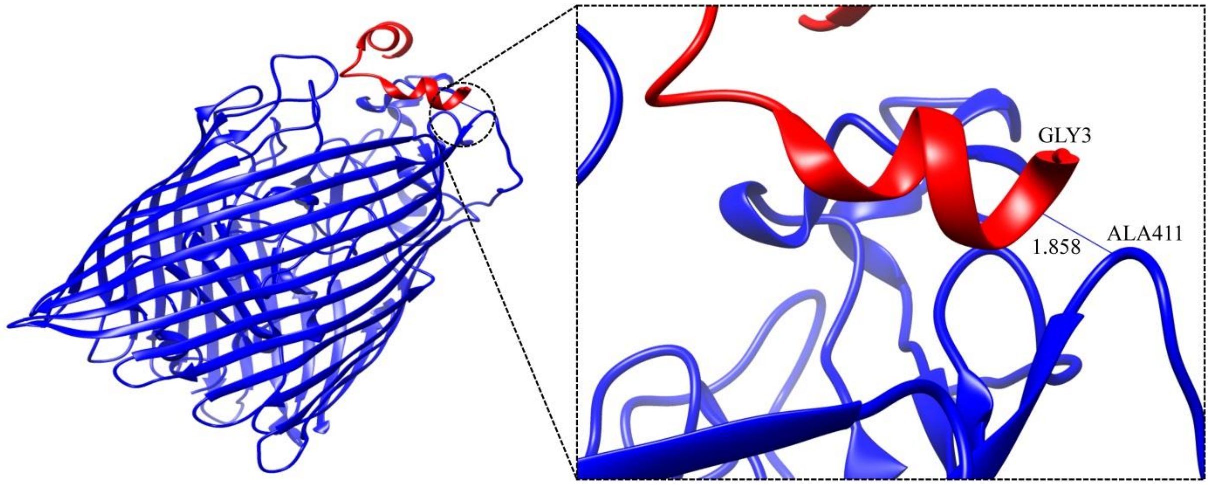

- Model FhuA-M6_A_2

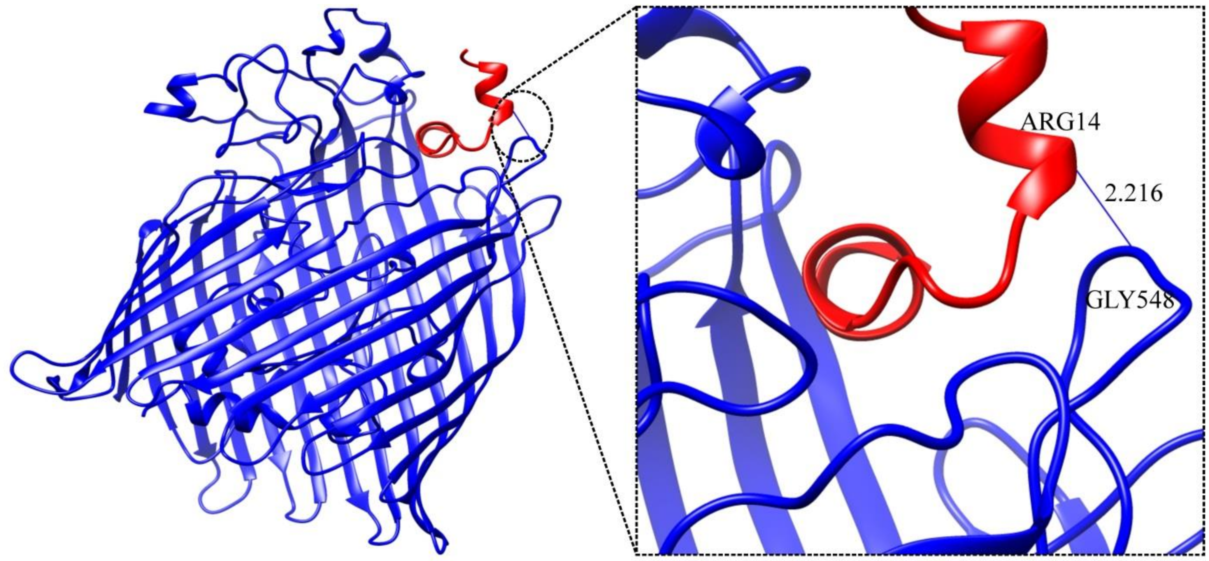

- Model FhuA-M6_A_3

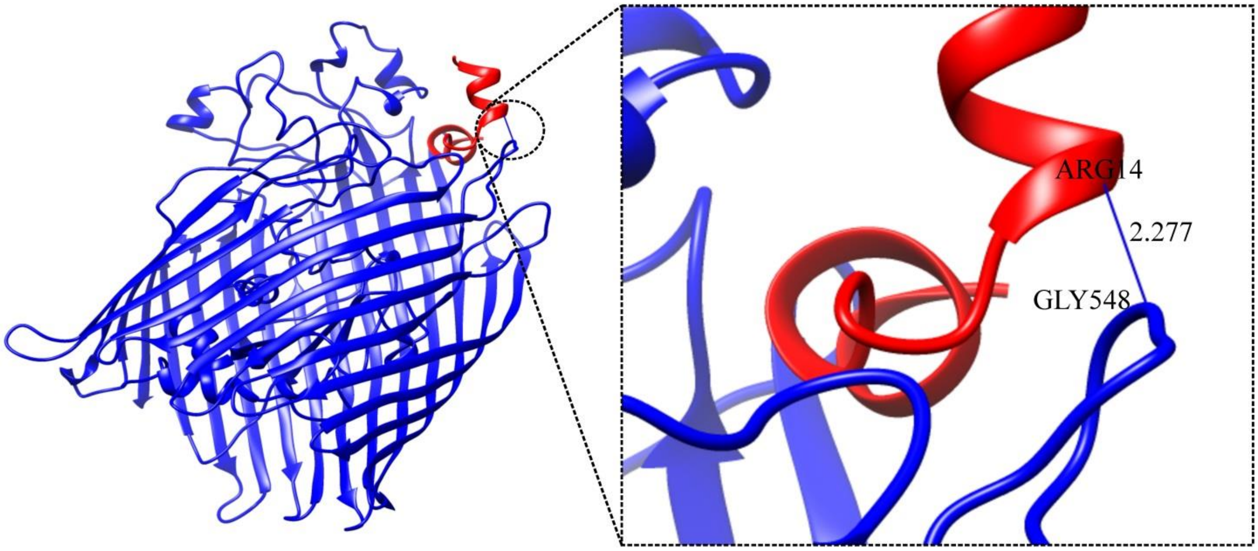

Model FhuA-M6_B

- Model FhuA-M6_B_1

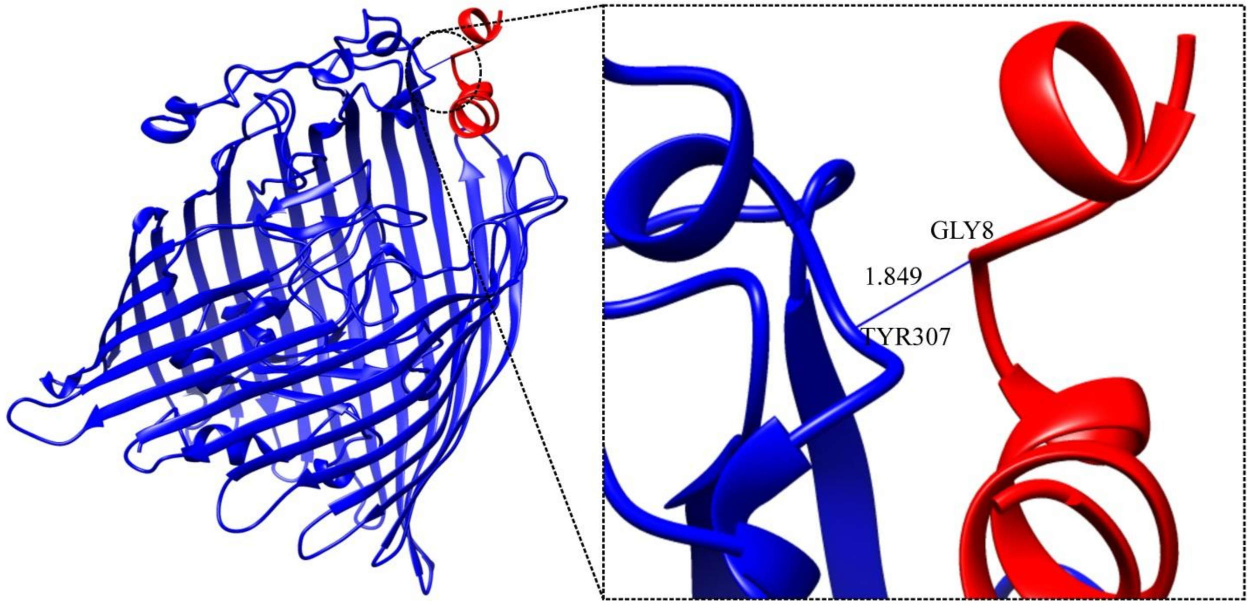

- Model FhuA-M6_B_2

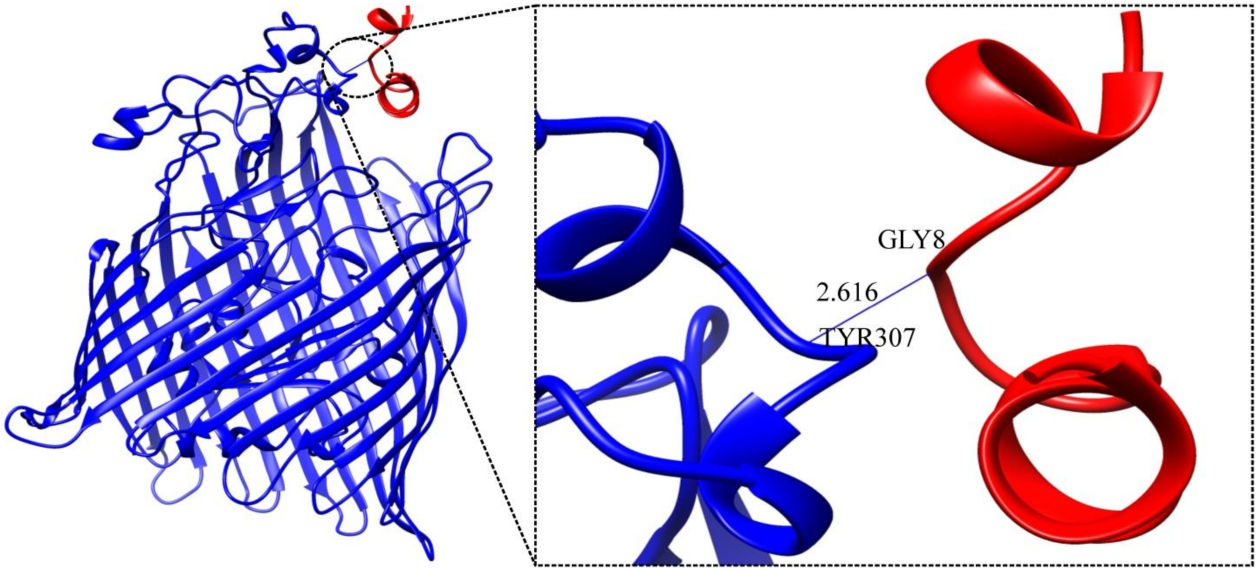

- Model FhuA-M6_B_3

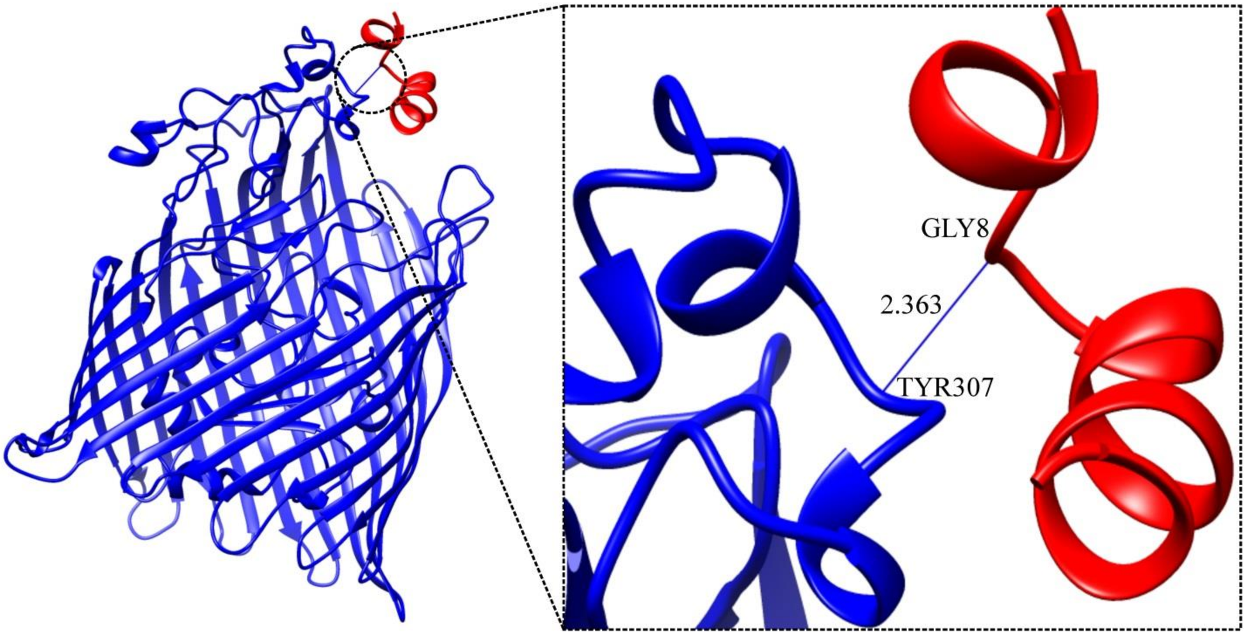

Model FhuA-M6_D

- Model FhuA-M6_D_1

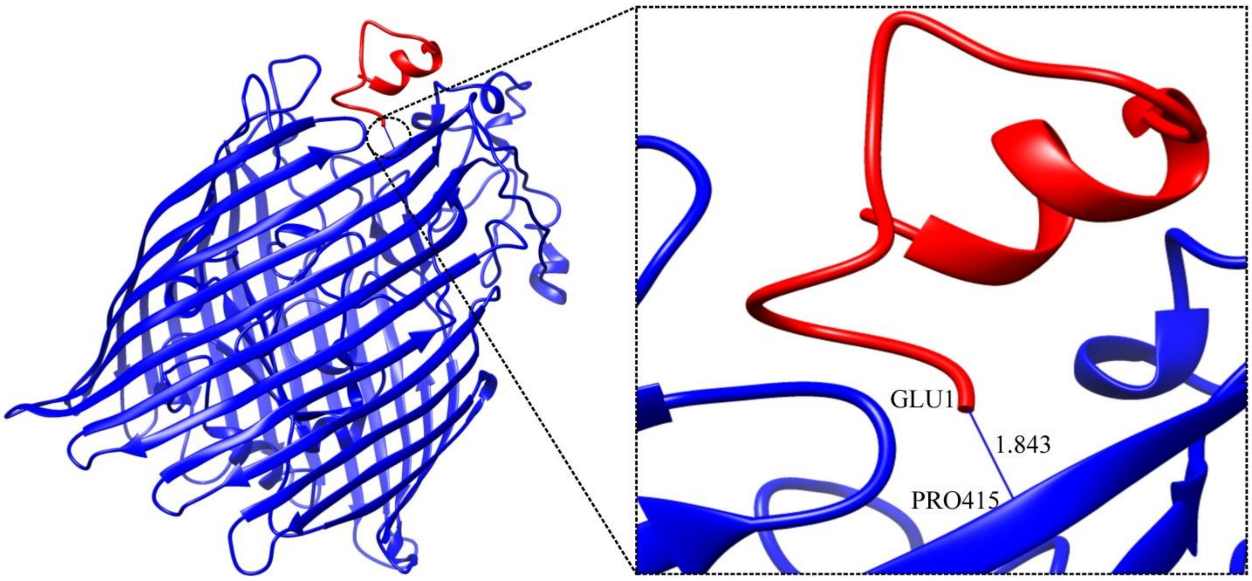

Images of the resulting complexes between LPS and Ib-M6 peptide models

Model LPS-M6_A

- Model LPS-M6_A_1

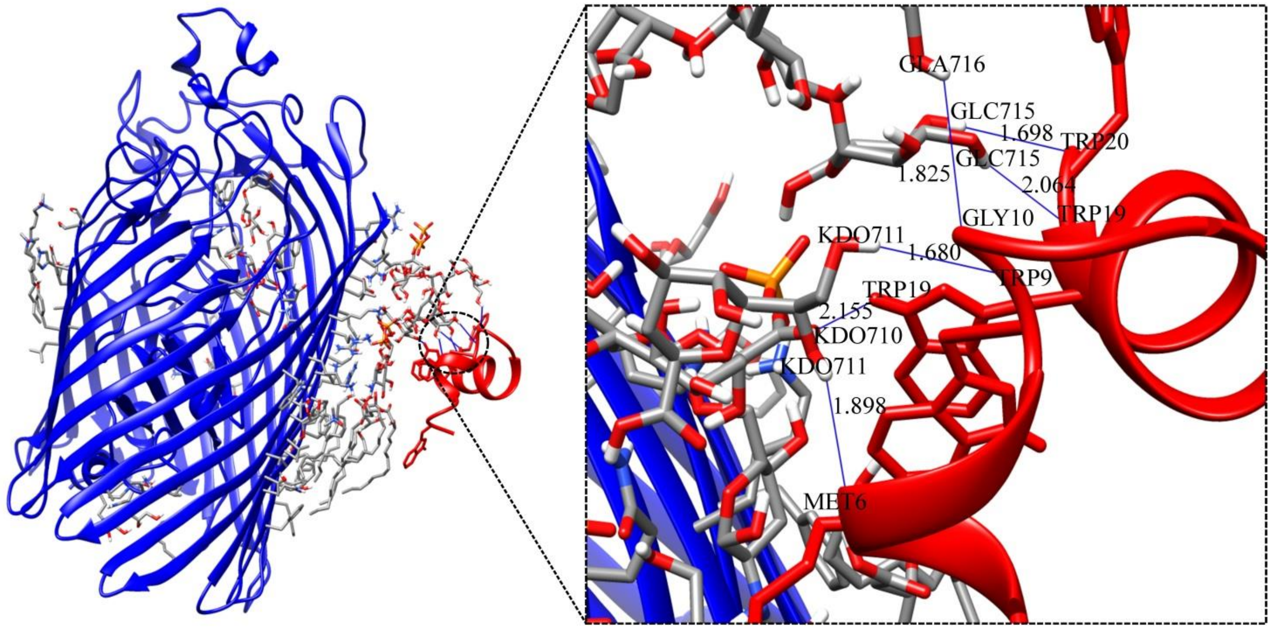

- Model LPS-M6_A_2

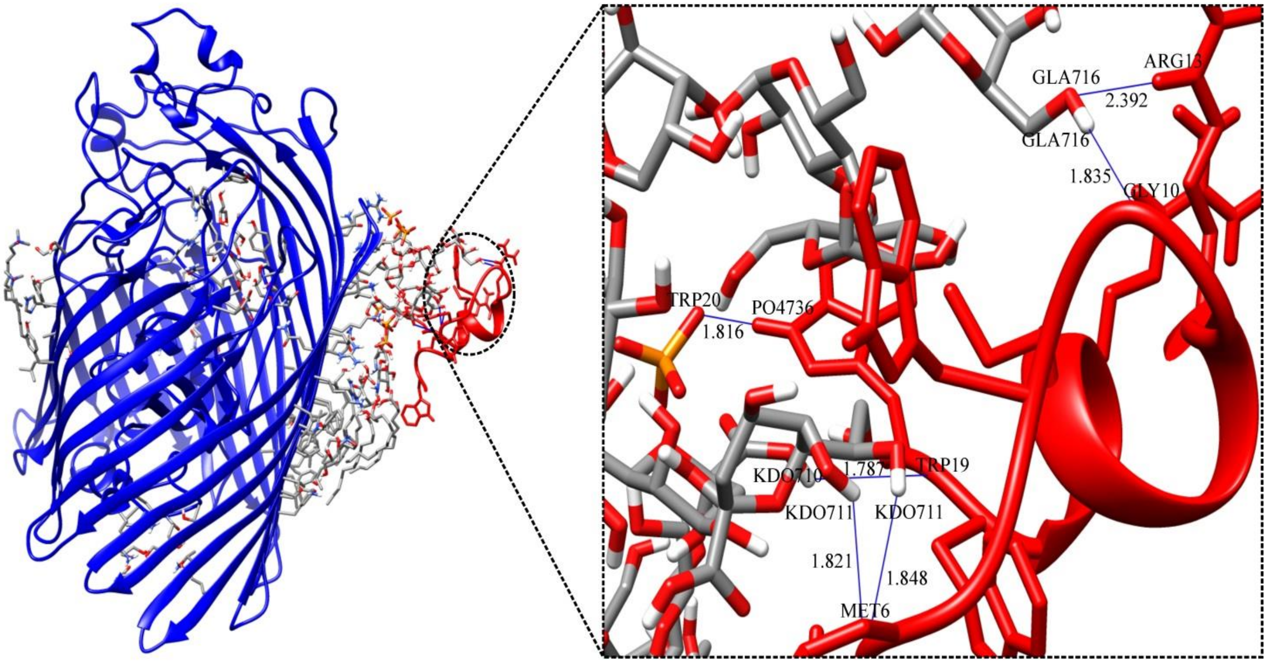

<u>Model LPS-M6_B</u>

- Model LPS-M6_B_1

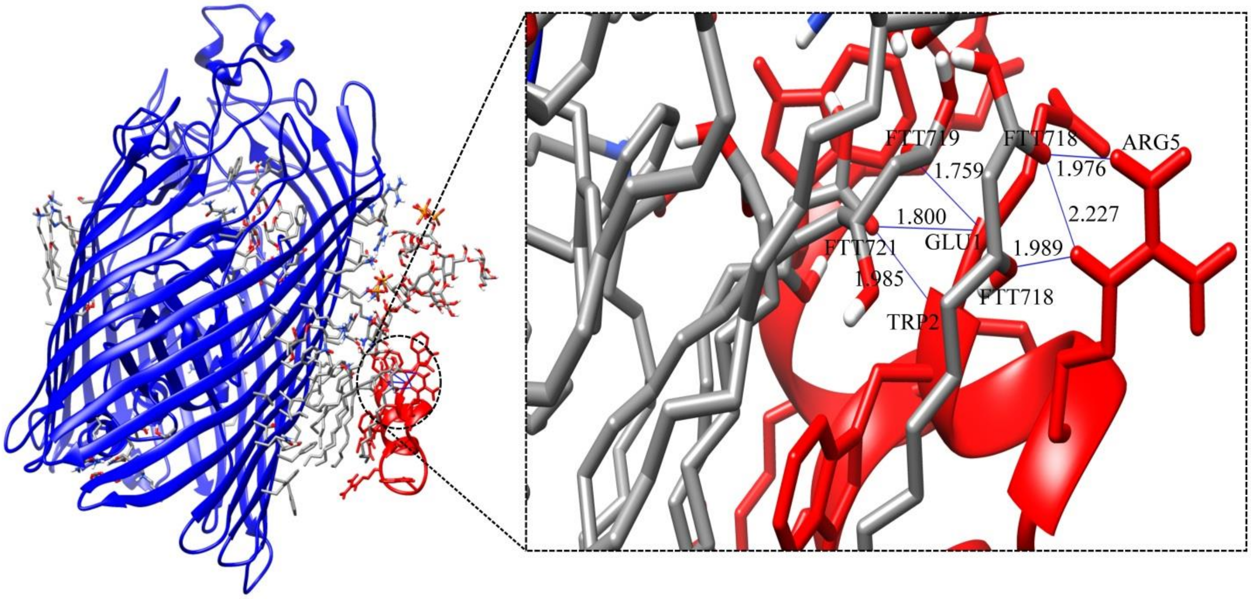

- Model LPS-M6_B_2

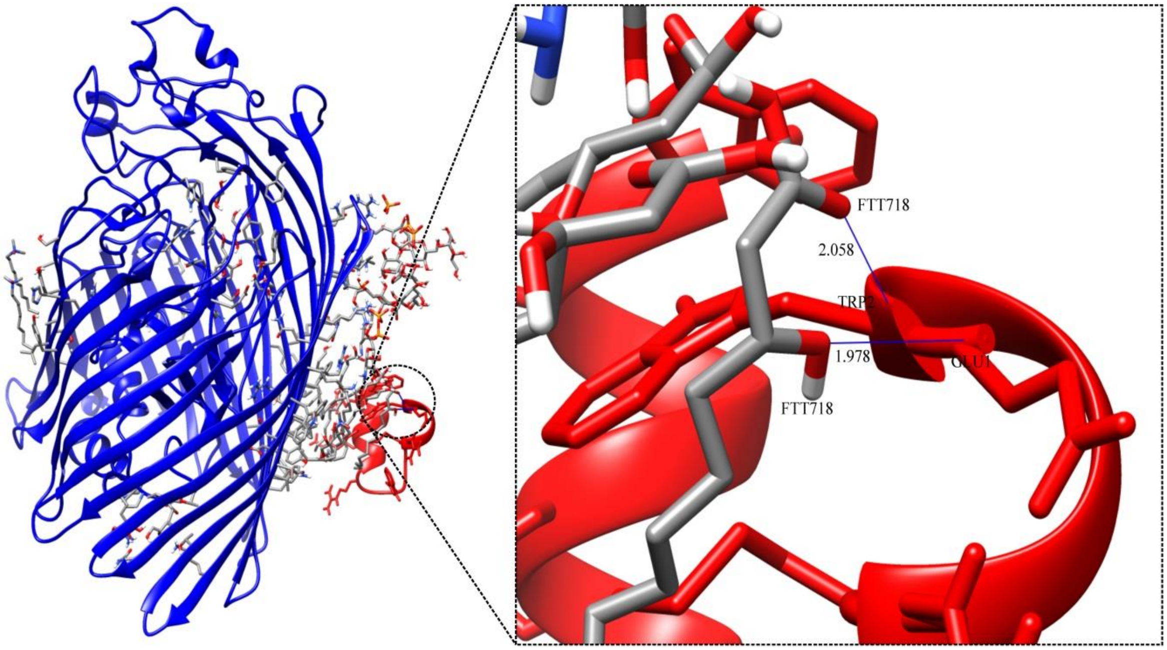

- Model LPS-M6_B_3

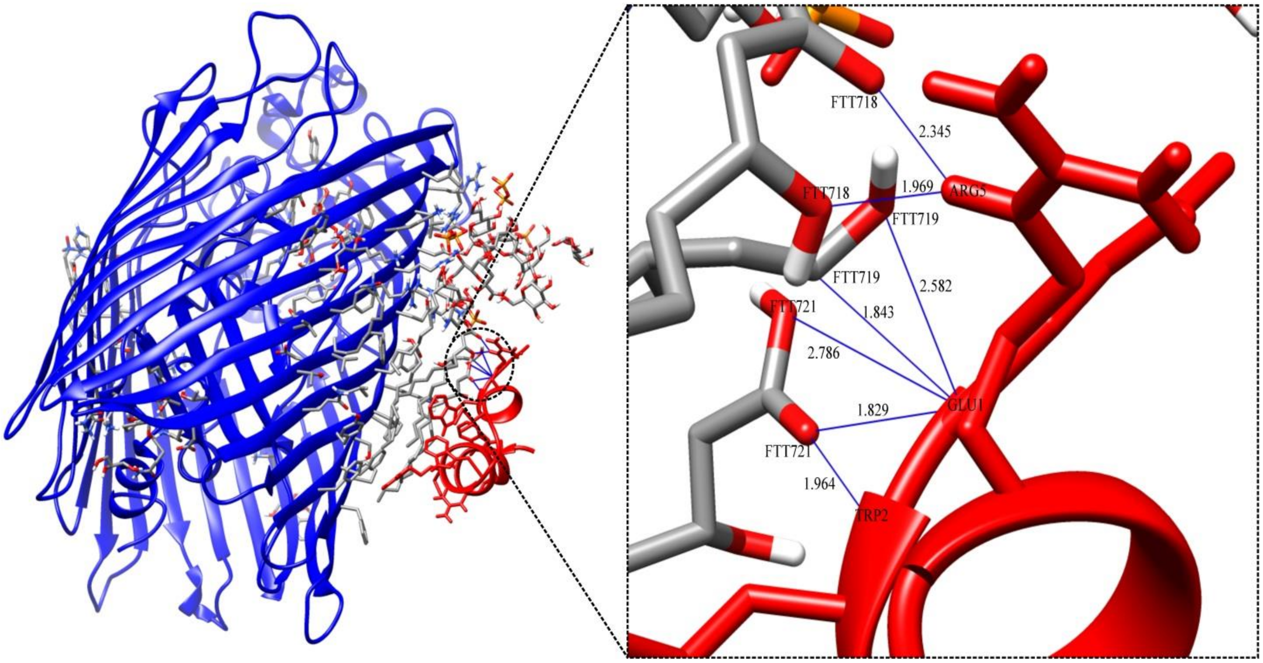

Model LPS-M6_C

- Model LPS_M6_C_2

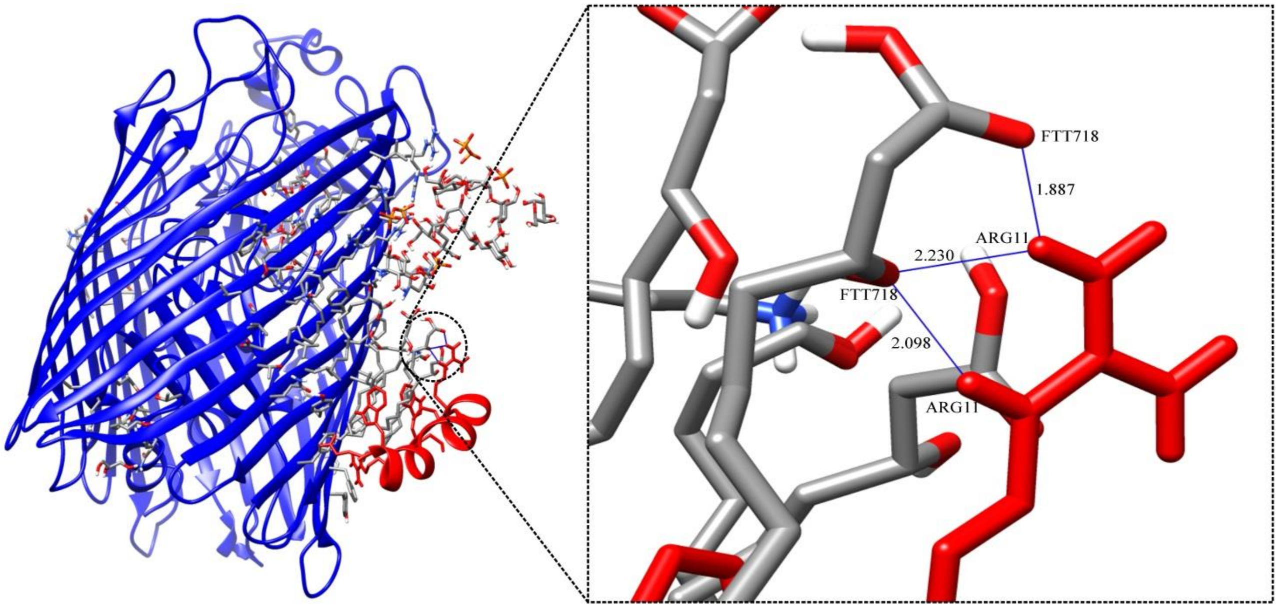

- Model LPS_M6_C_3

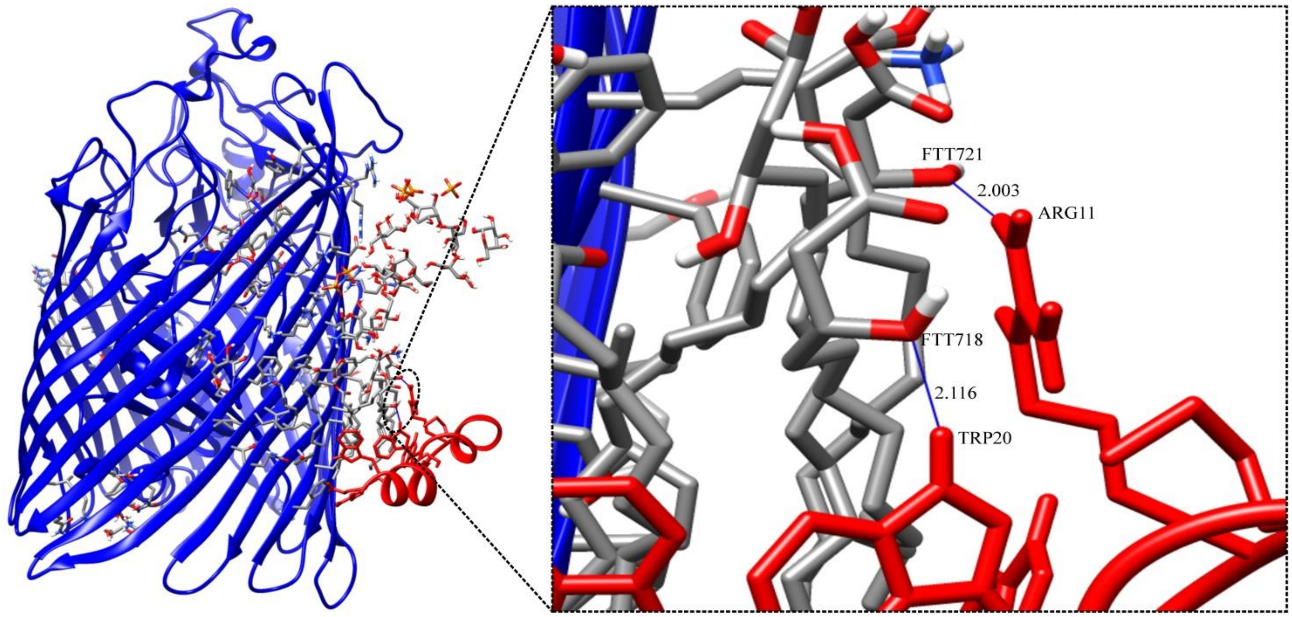

Model LPS-M6_D

- Model LPS_M6_D_1

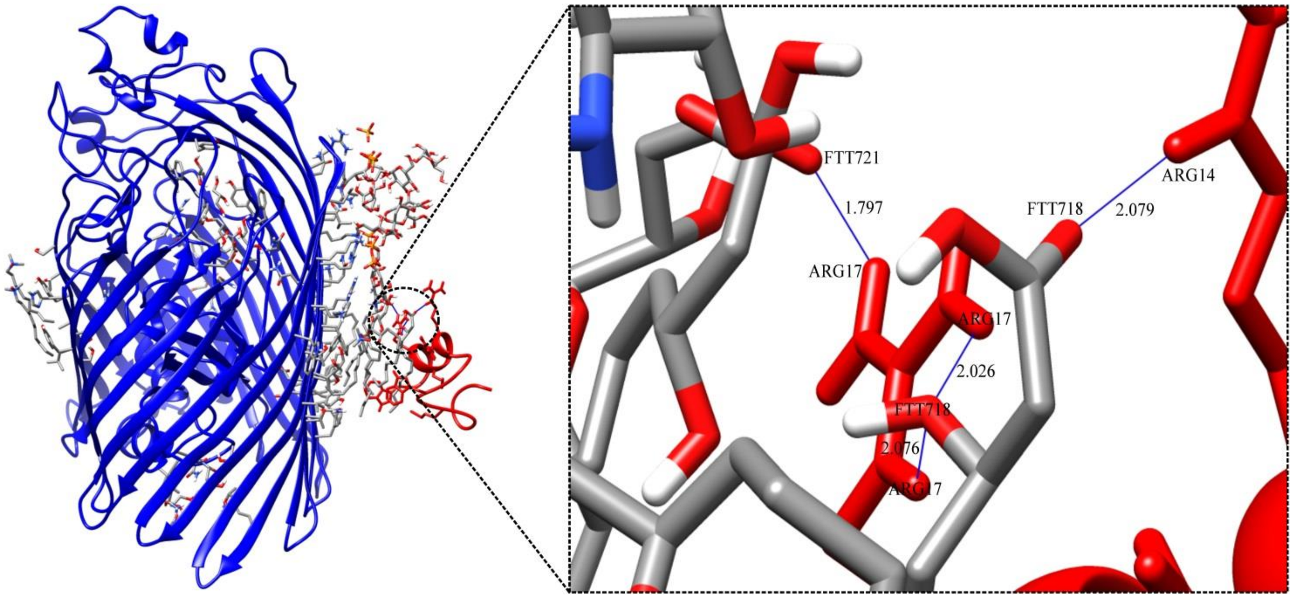

- Model LPS_M6_D_2

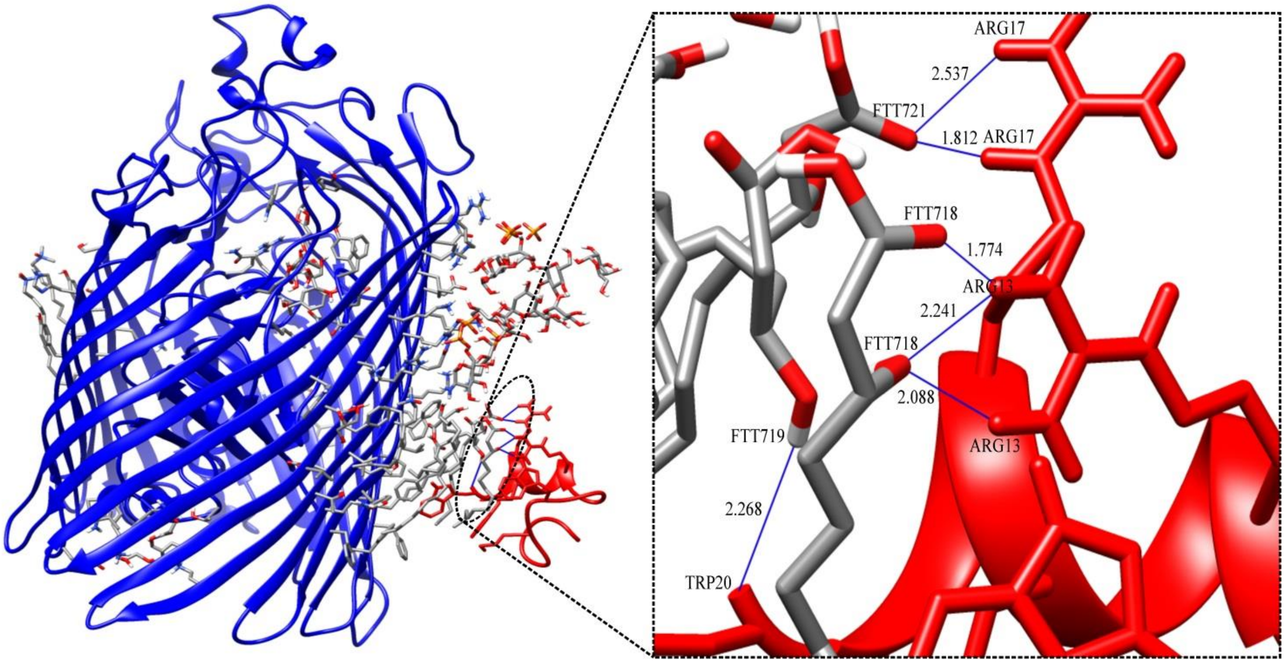

- Model LPS_M6_D_3

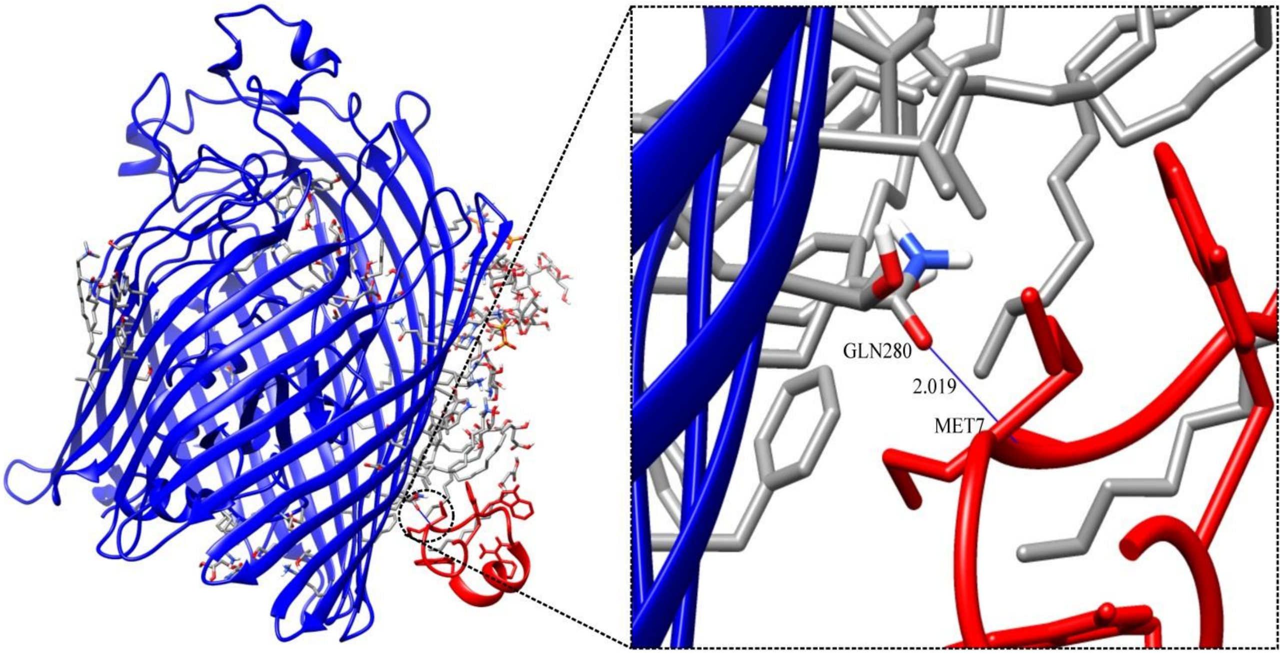

Model LPS-M6_E

- Model LPS_M6_E_1

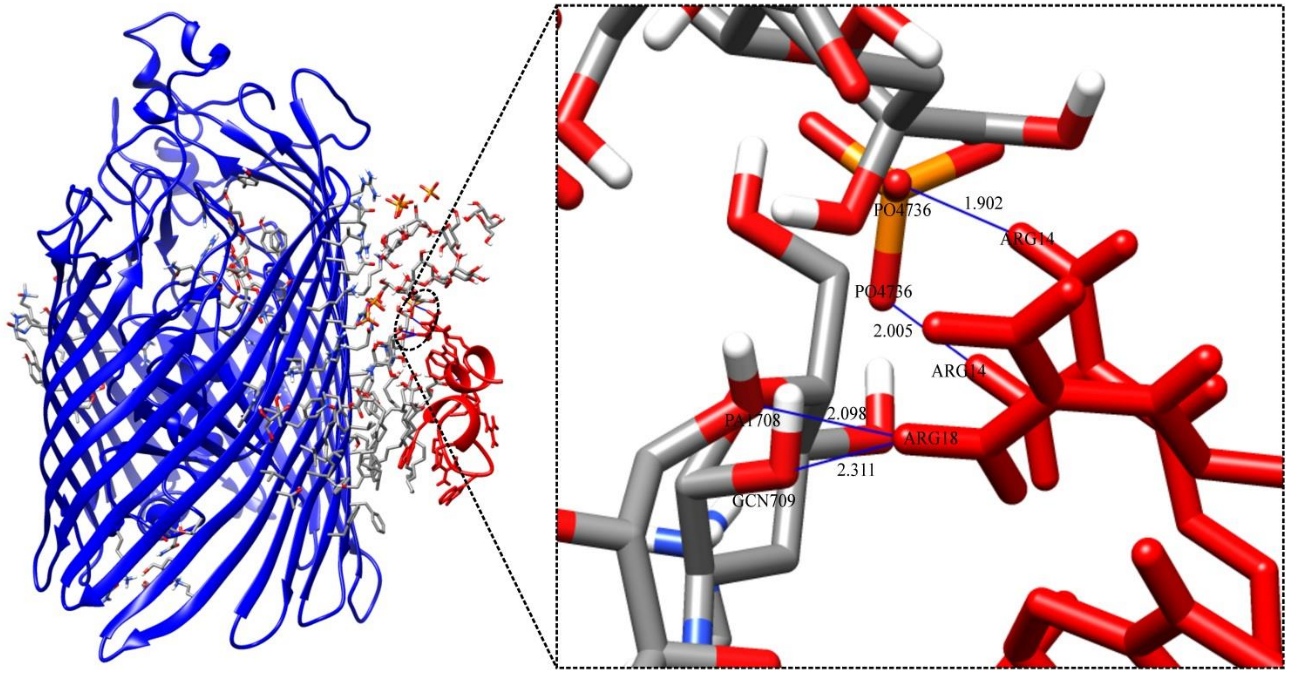

- Model LPS_M6_E_2

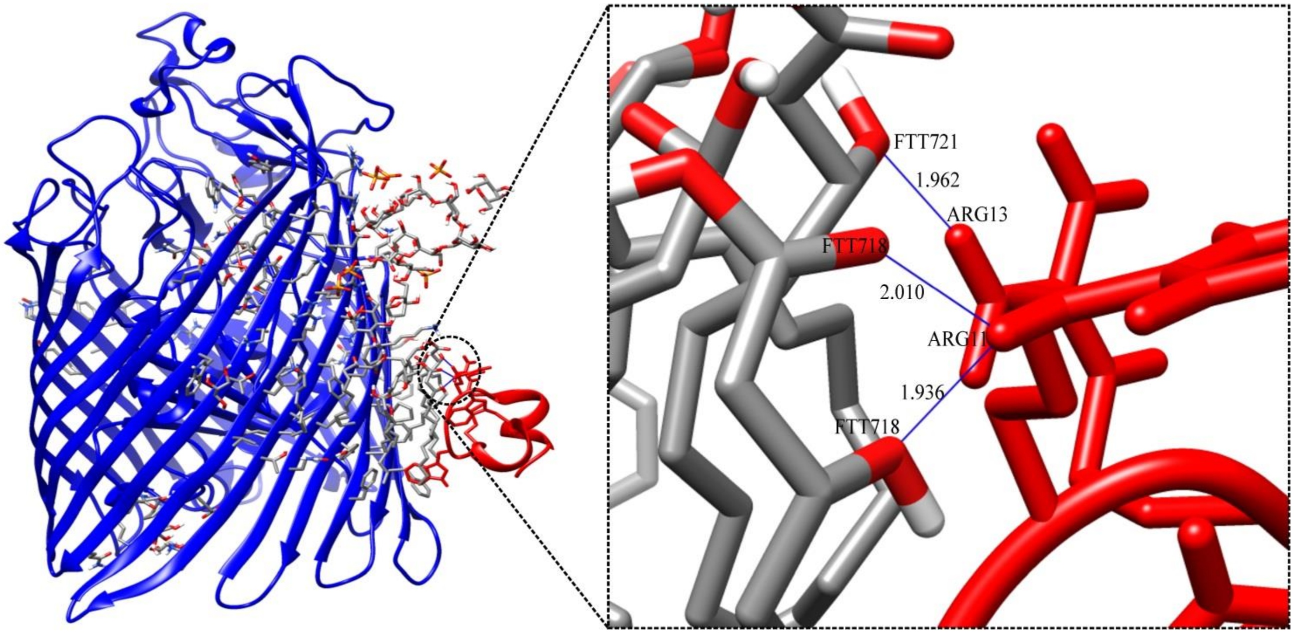

- Model LPS_M6_E_3

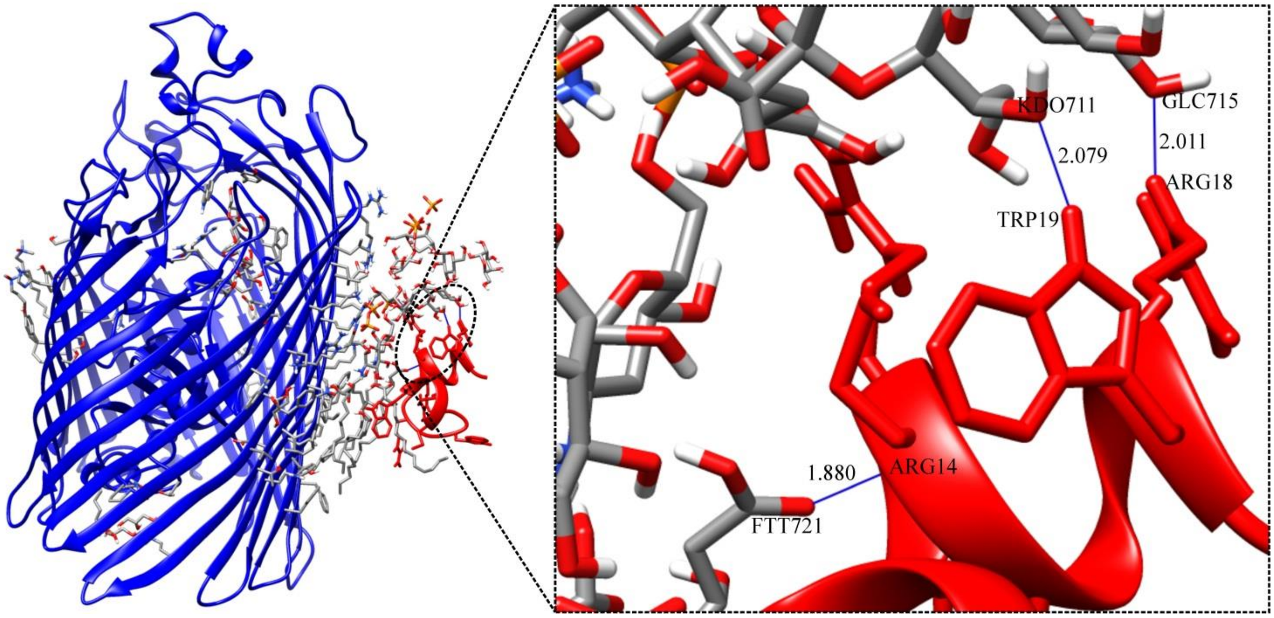

References

1 N. London, B. Raveh, E. Cohen, G. Fathi and O. Schueler-Furman, *Nucleic Acids Res.*, 2011, **39**, W249-53.
